# Validity of Administrative Data for Identifying Birth-Related Outcomes with the End Date of Pregnancy in a Japanese University Hospital

**DOI:** 10.3390/ijerph19084864

**Published:** 2022-04-16

**Authors:** Kentaro Tajima, Tomofumi Ishikawa, Fumiko Matsuzaki, Aoi Noda, Kei Morishita, Ryusuke Inoue, Noriyuki Iwama, Hidekazu Nishigori, Junichi Sugawara, Masatoshi Saito, Taku Obara, Nariyasu Mano

**Affiliations:** 1 Laboratory of Clinical Pharmacy, Graduate School of Pharmaceutical Sciences, Tohoku University, Sendai 980-8574, Japan; kentaro.tajima.s6@dc.tohoku.ac.jp (K.T.); tomofumi.ishikawa@pharm.hosp.tohoku.ac.jp (T.I.); mano@hosp.tohoku.ac.jp (N.M.); 2 Division of Preventive Medicine and Epidemiology, Tohoku Medical Megabank Organization, Tohoku University, Sendai 980-8573, Japan; fumiko.matsuzaki.b5@tohoku.ac.jp (F.M.); a.noda@megabank.tohoku.ac.jp (A.N.); noriyuki.iwama@med.tohoku.ac.jp (N.I.); 3 Department of Pharmaceutical Sciences, Tohoku University Hospital, Sendai 980-8574, Japan; kei.morishita.a7@tohoku.ac.jp; 4 Department of Molecular Epidemiology, Environment and Genome Research Center, Graduate School of Medicine, Tohoku University, Sendai 980-8573, Japan; 5 Department of Medical Informatics, Graduate School of Medicine, Tohoku University, Sendai 980-8574, Japan; rinoue@sic.med.tohoku.ac.jp; 6 Department of Obstetrics and Gynecology, Graduate School of Medicine, Tohoku University, Sendai 980-8574, Japan; masa_saito_lizard@yahoo.co.jp; 7 Fukushima Medical Center for Children and Women, Fukushima Medical University, Fukushima 960-1295, Japan; nishigo@fmu.ac.jp; 8 Division of Community Medical Supports, Tohoku Medical Megabank Organization, Tohoku University, Sendai 980-8573, Japan; jsugawara@med.tohoku.ac.jp; 9 Department of Feto-Maternal Medical Science, Environment and Genome Research Center, Graduate School of Medicine, Tohoku University, Sendai 980-8575, Japan

**Keywords:** administrative claims, Japan, obstetric delivery, positive predictive value, validation study

## Abstract

This study aimed to develop and validate claims-based algorithms for identifying live birth, fetal death, and cesarean section by utilizing administrative data from a university hospital in Japan. We included women who visited the Department of Obstetrics at a university hospital in 2018. The diagnosis, medical procedures, and medication data were used to identify potential cases of live birth, fetal death, and cesarean section. By reviewing electronic medical records, we evaluated the positive predictive values (PPVs) and the accuracy of the end date of pregnancy for each claims datum. “Selected algorithm 1” based on PPVs and “selected algorithm 2” based on both the PPVs and the accuracy of the end date of pregnancy were developed. A total of 1757 women were included, and the mean age was 32.8 years. The PPVs of “selected algorithm 1” and “selected algorithm 2” were both 98.1% for live birth, 99.0% and 98.9% for fetal death, and 99.7% and 100.0% for cesarean section, respectively. These findings suggest that the developed algorithms are useful for future studies for evaluating live birth, fetal death, and cesarean section with an accurate end date of pregnancy.

## 1. Introduction

The age of women giving birth has been rising in Japan [1]. Due to advanced maternal age, more women are likely to have chronic diseases, which require medication before or during pregnancy. For instance, it is now clear that cancer treatments, including anticancer medication, may cause organ damage, such as in the cardiovascular and endocrine systems [2,3,4,5,6,7,8,9]. Thus, evaluating birth outcomes for women with or after cancer medications is clinically important in real-world settings.

Utilizing the healthcare administrative database is one of the practical approaches to evaluating birth outcomes in real-world clinical settings and the information on rare treatment exposures [10,11,12]. Various administrative databases are currently available in Japan [13]; however, since administrative data are not collected for research purposes, the recorded information can sometimes result in incomplete and inaccurate data with misclassified outcomes. To provide a certain level of credibility for administrative data, evaluating the validity of the outcomes is crucial [14,15,16,17]. In addition, the regulatory agency in Japan, the United States, and Europe prepared the document to point out the importance of the outcome validation for the research use of administrative data [18,19,20]. Since the structure and contents of administrative claims and clinical practice differ among countries, a validation study conducted in Japan is necessary. Except for congenital malformation [21], the validity of birth-related outcomes has not been evaluated in Japan. Regarding the accuracy of the date of claim, a previous study in Japan reported the algorithms to determine the onset of pregnancy and delivery date [22]. However, the study population only included women who had given birth in the hospital, and the end date of pregnancy by specific birth-related outcomes was not assessed. Therefore, previous validation studies in Japan have been limited to addressing congenital malformation and the onset of pregnancy and delivery date without evaluating specific birth-related outcomes.

The current study bridges this research gap. It aims to develop and validate algorithms to identify birth-related outcomes (live birth, fetal death, and cesarean section) and the accuracy of the end date of pregnancy by using healthcare claims data from the Tohoku University Hospital in Japan.

## 2. Materials and Methods

### 2.1. Data Source and Study Population

We used electronic claims data at Tohoku University Hospital, containing diagnoses according to the International Classification of Diseases, 10th revision (ICD-10) codes, medical procedures (surgery and internal treatment), and medication. The study population included all women who visited the Department of Obstetrics at Tohoku University Hospital between January 2018 and December 2018. There were no exclusion criteria.

### 2.2. Definition and Identification of Birth-Related Outcomes

We evaluated live birth, fetal death, and cesarean section. In this study, we classified fetal death as miscarriage (defined as spontaneous abortion ≤ 21 weeks gestation), stillbirth (defined as spontaneous abortion > 21 weeks gestation), and induced abortion. To identify potential cases in each outcome, we used the obstetric diagnosis without the “suspected” flag, medication, and medical procedure listed in Table S1.

### 2.3. Review of the Potential Cases

We used electronic medical records (EMRs) as the gold standard to determine the accuracy of potential cases in each outcome. If a potential case had pregnancies several times in the study period based on EMR information, the accuracy was determined separately considering the date of the claim for each outcome. At Tohoku University Hospital, the Department of Obstetrics has developed and maintained a listing of EMR information regarding birth outcomes. We utilized the listing to review potential cases. However, since the listing only includes the birth outcomes of women who terminated their pregnancy after 12 weeks of gestation at Tohoku University, women who terminated their pregnancy before 12 weeks or those who delivered in other hospitals were not identified. Thus, if outcomes of potential cases were not available in the listing, the details of other unstructured data from EMRs were manually reviewed by three pharmacists. In the manual EMR review, two out of three pharmacists mutually reviewed the outcomes and the end date of pregnancy for each potential case. Any disagreements were resolved through discussion to obtain a final judgment by the third pharmacist.

### 2.4. Data Analysis

The age of the study population was calculated based on the first date of the claim entry and their birth date. However, if the women who visited the department of obstetrics at Tohoku University Hospital did not have any claims, their age at the time of their visit could not be identified directly. Therefore, we calculated the maternal age on 30 June 2018 because 30 June lies midway on the calendar.

To evaluate how accurately each claim datum identifies birth-related outcomes, we descriptively summarized the cases identified by each claim data. If the final outcomes could not be judged even after the EMR review due to a hospital transfer, these cases were categorized as “unevaluable”.

Additionally, we aimed to evaluate the accuracy of the date of the claim for birth-related outcomes. Regarding the end date of pregnancy in each birth-related outcome, we calculated the difference between the date of the claim data and that of the EMR. If one woman had multiple entries in the same claims data, we evaluated both the earliest date and the latest date and compared the results.

For the subsequent analysis, we took “selected algorithm 1” and “selected algorithm 2” to evaluate the validity of birth-related outcomes and the accuracy of the end date of pregnancy; “selected algorithm 1” was developed by combining the claims data (based on an “or” condition), of which positive predictive values (PPVs) were 80% or higher. PPVs were defined as the proportion of true-positive cases divided by the claims-positive cases. As a conservative approach for developing “selected algorithm 1”, we regarded “unevaluable” as a false positive instead of removing it from the dataset to avoid overestimating the accuracy. For exploratory analysis, we assessed the additional algorithms by subtracting those who had the claims codes of “selected algorithm 1” for fetal death from those who had inaccurate claims codes (with the PPVs under 80%) for live birth to evaluate whether the accuracy for live birth would improve.

From the “selected algorithm 1”, we developed a “selected algorithm 2” by strictly selecting the claims data with an accurate end date of pregnancy. We considered the date of the claim to be accurate if the percentage that met the criteria (the difference between the date of claim and the true end date of pregnancy was within ±7 days) was 80% or higher. For “selected algorithm 1” and “selected algorithm 2”, PPVs and corresponding 95% confidence intervals (CI) were also estimated.

All analyses were performed using SAS version 9.4 (SAS Institute, Inc., Cary, NC, USA).

## 3. Results

This study included 1757 women who visited the Department of Obstetrics at Tohoku University Hospital in 2018. Of them, 29 (1.7%) were 15–19 years old, 448 (25.5%) were 20–29 years old, 1089 (62.0%) were 30–39 years old, 186 (10.6%) were 40–49 years old, and 5 (0.3%) were over 50 years old. The mean age was 32.8 (standard deviation: 5.9) years.

Each claim entry and its name, the number of subjects, and the difference between the date of administrative data and EMRs for birth-related outcomes are summarized in Table 1 for live birth, Table 2 for fetal death, and Table 3 for cesarean section. All data were identified as true positives with PPVs of 80% or higher and were included in the selected algorithms in each outcome and subsequent analyses. Exceptions were some data for live birth in Table 1 (O711, rupture of the uterus during labor; O720, third-stage hemorrhage; O723, postpartum coagulation defects; J8001, uterine dilation and labor induction; methylergometrine maleate; gemeprost suppository) and fetal death in Table 2 (O011, complete and partial hydatidiform mole; O028, other specified abnormal products of conception). Regarding the large number of codes with PPVs of 80% or higher, oxytocin injection (*n* = 731), cesarean section (complexity fee addition) (K89800, *n* = 262), and cesarean section (elective cesarean section) (K89802, *n* = 175) were often observed for live birth (Table 1). Additionally, the surgical management of miscarriage (until 11 weeks) (K90901, *n* = 35), missed abortion (O021, *n* = 29), and dilatation and curettage (missed abortion) (K90920, *n* = 27) were common in fetal death (Table 2), while cesarean section (complexity fee addition) (K89800, *n* = 262), cesarean section (elective cesarean section) (K89802, *n* = 175), and cesarean section (emergency cesarean section) (K89801, *n* = 166) were common in cesarean sections (Table 3). It is worth noting that the number of codes (O601, preterm spontaneous labour with preterm delivery) was limited (*n* = 1).

For exploratory analysis, we subtracted those who had the claims data for fetal death from those who had the claims data with PPVs under 80% for live birth. The PPVs of live birth were 0.0% (O711), 79.2% (O720), 50.0% (O723), 76.2% (methylergometrine maleate injection), 81.2% (methylergometrine maleate tablet), and 0.0 % (gemeprost suppository), and no women were identified for J8001.

The date was generally accurate regarding all medical procedures with a PPV of 80% or higher for live birth, fetal death, and cesarean section. Considering the date difference between claims data and the end date of pregnancy in EMRs, the percentages that fell within the range of ±7 days were almost 100.0%, although K90920 (dilatation and curettage (missed abortion)) was 96.3%. However, regarding some claims of diagnosis, the percentages that fell within the range of ±7 days were under 80.0% for live birth (O680, labor and delivery complicated by fetal heart rate anomaly; O757, vaginal delivery following previous cesarean section; O820, delivery by elective cesarean section) in Table 1, fetal death (O009, ectopic pregnancy, unspecified; O021, missed abortion; O081, delayed or excessive haemorrhage following abortion and ectopic and molar pregnancy) in Table 2, and cesarean section (O820) in Table 3. Considering the date of diagnosis claims, the latest date was more accurate than the earliest date.

The PPVs of “selected algorithms 1” and “selected algorithms 2” were both 98.1% (95% CI 96.9–98.9%) for live birth, 99.0% (95% CI 94.4–100.0%) and 98.9% (95% CI 94.2–100.0%) for fetal death, and 99.7% (95% CI 98.4–100.0%) and 100.0% (95% CI 98.9–100.0%) for cesarean section, respectively (Table 4). Every algorithm, including “selected algorithm 1”, demonstrated the accuracy of the date, which showed that the percentages that fell within the range of ±7 days were over 80.0%.

For fetal death outcomes, only two cases resulted in stillbirth; thus, the table focusing on stillbirth has not been displayed. Miscarriage and induced abortion are summarized in Tables S2 and S3. Overall, although the potential cases in most claims data for identifying miscarriage and induced abortion were true-positive cases, K90901 (surgical management of miscarriage (until 11 weeks)) and K90902 (surgical management of miscarriage (from 12 weeks to 21 weeks)) were used for miscarriage instead of induced abortion; K90920 was used for induced abortion instead of miscarriage. The PPVs of “selected algorithms 1” and “selected algorithms 2” were 100.0% (95% CI 93.4–100.0%) and 100.0% (95% CI 92.9–100.0%) for miscarriage, and both were 89.2% (95% CI 74.6–97.0%) for induced abortion (Table S4).

## 4. Discussion

To the best of our knowledge, this is the first study to develop and evaluate the validity of algorithms to identify live birth, fetal death, including miscarriage and induced abortion, and cesarean section using administrative data in Japan.

The value of the current study lies in providing information on the accuracy of multiple birth-related outcomes based on claims information in Japan. In Western countries, various validation studies identifying live birth, miscarriage, stillbirth, induced abortion, and cesarean section have been reported and have demonstrated accuracy [23,24,25,26,27,28,29,30,31]. However, it is essential to conduct validation studies in Japan because some claims data in Japan are unique, and the procedures and treatment options might differ across countries. From a public health perspective, adverse obstetric outcomes affected by medication before or during pregnancy are of interest. Especially in the field of cancer, the improvement of patient survival outcomes through treatment progress has enabled cancer survivors to attempt childbearing [5]. In Taiwan, a study utilizing an administrative database revealed that cancer survivors had a higher risk of overall adverse birth outcomes and cesarean section [32]. It is expected that our findings will accelerate future studies in Japan to evaluate birth-related outcomes in the context of cancer treatment by using a claims-based administrative database.

In the current study, we developed an accurate algorithm for each birth-related outcome as “selected algorithm 1” by selecting the claims data with PPVs of 80% or higher. Combining the claims data could capture a larger number of potential outcomes than using the single claims data alone. Most of our investigated claims data related to fetal death and cesarean section represented accurate outcomes. However, some data (O711, O720, O723, J8001, methylergometrine maleate, and gemeprost suppository) resulted in inaccuracy for live birth and were not incorporated into “selected algorithm 1”. Considering this result, we assessed additional algorithms in the exploratory analysis by subtracting those who had the claims data of “selected algorithm 1” for fetal death from those who had these inaccurate claims data for live birth. Overall, although the PPV of methylergometrine maleate tablets was over 80% in this exploratory analysis, there was no remarkable improvement in the accuracy of live birth. Considering these exploratory results and the complexity of the algorithms, these claims codes might not be utilized for future studies to identify live births. Notably, the number of codes (O601, preterm spontaneous labour with preterm delivery) was very limited. Although preterm birth is an important adverse birth outcome, it might be difficult to identify it using the ICD-10 code in Japan. To identify preterm birth, the use of algorithms for the onset of pregnancy [22], as well as our algorithms of live birth, should be considered. To provide detailed information regarding fetal death, we also evaluated the fetal outcome as miscarriage and induced abortion separately. In general, although our planned claims codes were reasonable for identifying both outcomes, our results suggested that K90901 and K90902 were not used for induced abortion, and K90920 was not used for miscarriage.

Following the assessment of “selected algorithm 1”, we also assessed the accuracy of the end date of pregnancy; “selected algorithm 2” was identified as an accurate algorithm for both the outcome and its date. Considering the accuracy of the date, the medical procedure codes and the claims data by using the latest date were accurate in general, which was consistent with previous reports [22]. We recommend utilizing “selected algorithm 2” for each birth-related outcome for future studies that require an accurate outcome and its date. Considering the methodological point of view, we followed a step-by-step approach through the evaluation of (1) “single claims data”, (2) “selected algorithm 1 (combining the claims data with PPVs of 80% or higher)”, and (3) “selected algorithm 2 (combining both the claims data with PPVs of 80% or higher and the claims data with the accuracy of the end date of pregnancy (within ±7 days))”. Although claims data in Japan are unique and clinical or billing procedures might differ across countries, our approach to developing outcome algorithms would help in other countries.

Our study had some limitations. First, since the current study was conducted in a single university hospital in Japan, our results may not be generalizable to other medical institutions, including general clinics. The population in this study likely included older women, who may be at risk during pregnancy and delivery. Furthermore, the number of fetal deaths and cesarean sections might be larger in university hospitals than in general clinics. Although the system for medical fee processing was standardized and the government provided the master files under the healthcare system in Japan, further studies may be required to determine whether our results could be extrapolated to other hospitals. Second, we were unable to review the negative cases of the algorithm due to the study’s resources. Thus, the sensitivity, specificity, and negative predictive value were not assessed. We believe that our results provide useful information, especially for future comparative studies, to evaluate the relative risk of birth-related outcomes between exposure and control. Third, regarding birth-related outcomes, we assessed outcomes for women who had transferred to another hospital as “unevaluable”. However, as a conservative approach, we defined the selected algorithms in the current study by regarding “unevaluable” as false positives instead of removing them from the dataset to avoid overestimating the accuracy. Lastly, we could not evaluate some claims data related to birth-related outcomes because some specific codes were not observed in the administrative claims during our study period. For example, our study did not cover some of the ICD-10 codes of “O00-O99 Pregnancy, childbirth, and the puerperium”. Therefore, the accuracy of non-evaluated claims data was uncertain. Furthermore, given that some claims data were based on only a few potential cases in this study, results with these uncommon claims data should be carefully interpreted. Therefore, future studies with these non-evaluated and uncommon claims data in our study are suggested.

## 5. Conclusions

We developed reliable combination algorithms to identify live birth, fetal death, and cesarean section based on the evaluation of the accuracy of each claim data, as well as the accuracy of the date of claim. Since validated claims-based algorithms with information on the PPVs and the accuracy of the dates are required to provide a certain level of credibility for the research use of claims data, we recommend utilizing our validated algorithms for each birth-related outcome. Our findings will benefit future studies that attempt to evaluate birth-related outcomes in real-world clinical settings.

## Figures and Tables

**Table 1:** Accuracy of claims data to identify live birth and the difference between the date of claim data and the end date of pregnancy in EMRs.

						Relative Difference (The Date of Claim Data Minus the End Date of Pregnancy in EMRs) (d)				
		N of Subjects				0 Days	Within 3 Days	Within 7 Days	Within 14 Days	Within 30 Days
Code	Code Name	Claims	TP (%)	FP (%)	Unevaluable (%)	n (%) ^a^				
O601	Preterm spontaneous labor with preterm delivery	1	1 (100.0)	0 (0.0)	0 (0.0)	1 (100.0)	1 (100.0)	1 (100.0)	1 (100.0)	1 (100.0)
O624	Hypertonic, incoordinate, and prolonged uterine contractions	9	9 (100.0)	0 (0.0)	0 (0.0)	8 (88.9)	9 (100.0)	9 (100.0)	9 (100.0)	9 (100.0)
O640	Obstructed labor due to incomplete rotation of fetal head	1	1 (100.0)	0 (0.0)	0 (0.0)	1 (100.0)	1 (100.0)	1 (100.0)	1 (100.0)	1 (100.0)
O654	Obstructed labor due to fetopelvic disproportion, unspecified	9	9 (100.0)	0 (0.0)	0 (0.0)	9 (100.0)	9 (100.0)	9 (100.0)	9 (100.0)	9 (100.0)
O655	Obstructed labor due to abnormality of maternal pelvic organs	1	1 (100.0)	0 (0.0)	0 (0.0)	1 (100.0)	1 (100.0)	1 (100.0)	1 (100.0)	1 (100.0)
O669	Obstructed labor, unspecified	6	6 (100.0)	0 (0.0)	0 (0.0)	6 (100.0)	6 (100.0)	6 (100.0)	6 (100.0)	6 (100.0)
O680	Labor and delivery complicated by fetal heart rate anomaly	3	3 (100.0)	0 (0.0)	0 (0.0)	1 (33.3)	1 (33.3)	2 (66.7)	2 (66.7)	3 (100.0)
O683	Labor and delivery complicated by biochemical evidence of fetal stress	28	28 (100.0)	0 (0.0)	0 (0.0)	27 (96.4)	27 (96.4)	28 (100.0)	28 (100.0)	28 (100.0)
O690	Labor and delivery complicated by prolapse of cord	1	1 (100.0)	0 (0.0)	0 (0.0)	0 (0.0)	1 (100.0)	1 (100.0)	1 (100.0)	1 (100.0)
O700	First degree perineal laceration during delivery	1	1 (100.0)	0 (0.0)	0 (0.0)	1 (100.0)	1 (100.0)	1 (100.0)	1 (100.0)	1 (100.0)
O711	Rupture of uterus during labor	1	0 (0.0)	1 (100.0)	0 (0.0)	0 (0.0)	0 (0.0)	0 (0.0)	0 (0.0)	0 (0.0)
O717	Obstetric hematoma of pelvis	5	5 (100.0)	0 (0.0)	0 (0.0)	4 (80.0)	5 (100.0)	5 (100.0)	5 (100.0)	5 (100.0)
O720	Third-stage hemorrhage	27	19 (70.4)	8 (29.6)	0 (0.0)	8 (42.1)	9 (47.4)	10 (52.6)	11 (57.9)	13 (68.4)
O721	Other immediate postpartum hemorrhage	91	88 (96.7)	1 (1.1)	2 (2.2)	82 (93.2)	87 (98.9)	87 (98.9)	87 (98.9)	88 (100.0)
O723	Postpartum coagulation defects	2	1 (50.0)	1 (50.0)	0 (0.0)	1 (100.0)	1 (100.0)	1 (100.0)	1 (100.0)	1 (100.0)
O757	Vaginal delivery following previous cesarean section (the earliest date) ^b^	20	20 (100.0)	0 (0.0)	0 (0.0)	0 (0.0)	0 (0.0)	4 (20.0)	13 (65.0)	20 (100.0)
O757	Vaginal delivery following previous cesarean section (the latest date) ^b^	20	20 (100.0)	0 (0.0)	0 (0.0)	1 (5.0)	1 (5.0)	5 (25.0)	14 (70.0)	20 (100.0)
O800	Spontaneous vertex delivery	74	74 (100.0)	0 (0.0)	0 (0.0)	65 (87.8)	70 (94.6)	74 (100.0)	74 (100.0)	74 (100.0)
O820	Delivery by elective cesarean section (the earliest date) ^b^	16	16 (100.0)	0 (0.0)	0 (0.0)	3 (18.8)	4 (25.0)	6 (37.5)	11 (68.8)	15 (93.8)
O820	Delivery by elective cesarean section (the latest date) ^b^	16	16 (100.0)	0 (0.0)	0 (0.0)	3 (18.8)	5 (31.3)	7 (43.8)	12 (75.0)	15 (93.8)
K89300	Vacuum extractions	101	101 (100.0)	0 (0.0)	0 (0.0)	101 (100.0)	101 (100.0)	101 (100.0)	101 (100.0)	101 (100.0)
K89601	Suture of perineal laceration during delivery (muscle layer)	1	1 (100.0)	0 (0.0)	0 (0.0)	1 (100.0)	1 (100.0)	1 (100.0)	1 (100.0)	1 (100.0)
K89603	Suture of perineal laceration during delivery (vaginal fornix)	1	1 (100.0)	0 (0.0)	0 (0.0)	1 (100.0)	1 (100.0)	1 (100.0)	1 (100.0)	1 (100.0)
K89800	Cesarean section (complexity fee addition)	262	257 (98.1)	5 (1.9)	0 (0.0)	255 (99.2)	257 (100.0)	257 (100.0)	257 (100.0)	257 (100.0)
K89801	Cesarean section (emergency cesarean section)	166	162 (97.6)	4 (2.4)	0 (0.0)	160 (98.8)	162 (100.0)	162 (100.0)	162 (100.0)	162 (100.0)
K89802	Cesarean section (elective cesarean section)	175	175 (100.0)	0 (0.0)	0 (0.0)	175 (100.0)	175 (100.0)	175 (100.0)	175 (100.0)	175 (100.0)
K90400	Obstetric hysterectomy (Porro’s operation)	5	5 (100.0)	0 (0.0)	0 (0.0)	5 (100.0)	5 (100.0)	5 (100.0)	5 (100.0)	5 (100.0)
J8001	Uterine dilation and labor induction (laminaria) (the earliest date) ^b^	6	0 (0.0)	6 (100.0)	0 (0.0)	0 (0.0)	0 (0.0)	0 (0.0)	0 (0.0)	0 (0.0)
J8001	Uterine dilation and labor induction (laminaria) (the latest date) ^b^	6	0 (0.0)	6 (100.0)	0 (0.0)	0 (0.0)	0 (0.0)	0 (0.0)	0 (0.0)	0 (0.0)
N/A	Oxytocin injection (the earliest date) ^b^	731	717 (98.1)	13 (1.8)	1 (0.1)	616 (85.9)	711 (99.2)	714 (99.6)	717 (100.0)	717 (100.0)
N/A	Oxytocin injection (the latest date) ^b^	731	717 (98.1)	13 (1.8)	1 (0.1)	552 (77.0)	711 (99.2)	711 (99.2)	715 (99.7)	716 (99.9)
N/A	Methylergometrine maleate injection (the earliest date) ^b^	24	16 (66.7)	7 (29.2)	1 (4.2)	14 (87.5)	15 (93.8)	15 (93.8)	16 (100.0)	16 (100.0)
N/A	Methylergometrine maleate injection (the latest date) ^b^	24	16 (66.7)	7 (29.2)	1 (4.2)	13 (81.3)	14 (87.5)	15 (93.8)	16 (100.0)	16 (100.0)
N/A	Dinoprost injection (the earliest date) ^b^	7	7 (100.0)	0 (0.0)	0 (0.0)	3 (42.9)	6 (85.7)	7 (100.0)	7 (100.0)	7 (100.0)
N/A	Dinoprost injection (the latest date) ^b^	7	7 (100.0)	0 (0.0)	0 (0.0)	3 (42.9)	6 (85.7)	7 (100.0)	7 (100.0)	7 (100.0)
N/A	Gemeprost suppository (the earliest date) ^b^	27	0 (0.0)	27 (100.0)	0 (0.0)	0 (0.0)	0 (0.0)	0 (0.0)	0 (0.0)	0 (0.0)
N/A	Gemeprost suppository (the latest date) ^b^	27	0 (0.0)	27 (100.0)	0 (0.0)	0 (0.0)	0 (0.0)	0 (0.0)	0 (0.0)	0 (0.0)
N/A	Methylergometrine maleate tablet (the earliest date) ^b^	218	140 (64.2)	78 (35.8)	0 (0.0)	3 (2.1)	7 (5.0)	31 (22.1)	39 (27.9)	87 (62.1)
N/A	Methylergometrine maleate tablet (the latest date) ^b^	218	140 (64.2)	78 (35.8)	0 (0.0)	1 (0.7)	4 (2.9)	24 (17.1)	32 (22.9)	71 (50.7)

Abbreviations: EMRs, electronic medical records; TP, true positive; FP, false positive. ^a^ Regarding the relative difference, cases that were both true positive and available for “the end date of pregnancy in EMRs” are shown. Therefore, some numbers could have discrepancies between the number of true-positive cases and the numbers in the rows of the relative difference if some cases missed the end date of pregnancy in EMRs, even though the EMR outcome itself was identified. ^b^ Existing multiple entries in the same claim codes for the same women; both the earliest and the latest dates were analyzed.

**Table 2:** Accuracy of claims data to identify fetal death ^a^ and the difference between the date of claim data and the end date of pregnancy in EMRs.

						Relative Difference (The Date of Claim Data Minus the End Date of Pregnancy in EMRs) (d)				
		N of Subjects				0 Days	Within 3 Days	Within 7 Days	Within 14 Days	Within 30 Days
Code	Code Name	Claims	TP (%)	FP (%)	Unevaluable (%)	n (%) ^b^				
O008	Other ectopic pregnancy	1	1 (100.0)	0 (0.0)	0 (0.0)	0 (0.0)	0 (0.0)	1 (100.0)	1 (100.0)	1 (100.0)
O009	Ectopic pregnancy, unspecified	2	2 (100.0)	0 (0.0)	0 (0.0)	0 (0.0)	0 (0.0)	1 (50.0)	1 (50.0)	2 (100.0)
O010	Classical hydatidiform mole (the earliest date) ^c^	4	4 (100.0)	0 (0.0)	0 (0.0)	1 (25.0)	4 (100.0)	4 (100.0)	4 (100.0)	4 (100.0)
O010	Classical hydatidiform mole (the latest date) ^c^	4	4 (100.0)	0 (0.0)	0 (0.0)	1 (25.0)	3 (75.0)	4 (100.0)	4 (100.0)	4 (100.0)
O011	Incomplete and partial hydatidiform mole	1	0 (0.0)	1 (100.0)	0 (0.0)	0 (0.0)	0 (0.0)	0 (0.0)	0 (0.0)	0 (0.0)
O021	Missed abortion	29	29 (100.0)	0 (0.0)	0 (0.0)	10 (35.7)	16 (57.1)	22 (78.6)	28 (100.0)	28 (100.0)
O028	Other specified abnormal products of conception	1	0 (0.0)	1 (100.0)	0 (0.0)	0 (0.0)	0 (0.0)	0 (0.0)	0 (0.0)	0 (0.0)
O034	Incomplete spontaneous abortion without complication	2	2 (100.0)	0 (0.0)	0 (0.0)	2 (100.0)	2 (100.0)	2 (100.0)	2 (100.0)	2 (100.0)
O039	Complete or unspecified spontaneous abortion without complication	5	5 (100.0)	0 (0.0)	0 (0.0)	3 (75.0)	4 (100.0)	4 (100.0)	4 (100.0)	4 (100.0)
O049	Medical abortion, complete or unspecified, without complication (the earliest date) ^c^	22	21 (95.5)	0 (0.0)	1 (4.5)	7 (33.3)	15 (71.4)	17 (81.0)	20 (95.2)	21 (100.0)
O049	Medical abortion, complete or unspecified, without complication (the latest date) ^c^	22	21 (95.5)	0 (0.0)	1 (4.5)	8 (38.1)	17 (81.0)	19 (90.5)	21 (100.0)	21 (100.0)
O080	Genital tract and pelvic infection following abortion and ectopic and molar pregnancy	1	1 (100.0)	0 (0.0)	0 (0.0)	1 (100.0)	1 (100.0)	1 (100.0)	1 (100.0)	1 (100.0)
O081	Delayed or excessive haemorrhage following abortion and ectopic and molar pregnancy	1	1 (100.0)	0 (0.0)	0 (0.0)	0 (0.0)	0 (0.0)	0 (0.0)	0 (0.0)	1 (100.0)
O364	Maternal care for intrauterine death (the earliest date) ^c^	8	8 (100.0)	0 (0.0)	0 (0.0)	1 (12.5)	2 (25.0)	7 (87.5)	8 (100.0)	8 (100.0)
O364	Maternal care for intrauterine death (the latest date) ^c^	8	8 (100.0)	0 (0.0)	0 (0.0)	2 (25.0)	3 (37.5)	8 (100.0)	8 (100.0)	8 (100.0)
P95	Fetal death of unspecified cause	1	1 (100.0)	0 (0.0)	0 (0.0)	1 (100.0)	1 (100.0)	1 (100.0)	1 (100.0)	1 (100.0)
K90901	Surgical management of miscarriage (until 11 weeks)	35	35 (100.0)	0 (0.0)	0 (0.0)	35 (100.0)	35 (100.0)	35 (100.0)	35 (100.0)	35 (100.0)
K90902	Surgical management of miscarriage (from 12 weeks to 21 weeks)	1	1 (100.0)	0 (0.0)	0 (0.0)	1 (100.0)	1 (100.0)	1 (100.0)	1 (100.0)	1 (100.0)
K90920	Dilatation and curettage (missed abortion)	27	27 (100.0)	0 (0.0)	0 (0.0)	26 (96.3)	26 (96.3)	26 (96.3)	26 (96.3)	26 (96.3)
K91100	Removal of hydatidiform mole	6	6 (100.0)	0 (0.0)	0 (0.0)	6 (100.0)	6 (100.0)	6 (100.0)	6 (100.0)	6 (100.0)

Abbreviations: EMRs, electronic medical records; TP, true positive; FP, false positive. ^a^ The results of miscarriage and induced abortion; individual components of fetal death in this study are shown in Tables S2 and S3. ^b.^ Regarding the relative difference, cases that were both true positive and available for “the end date of pregnancy in EMRs” are shown. Therefore, some numbers could have discrepancies between the number of true-positive cases and the numbers in the rows of the relative difference if some cases missed the end date of pregnancy in EMRs, even though the EMR outcome itself was identified. ^c^ Existing multiple entries in the same claims code for the same women; both the earliest and the latest dates were analyzed.

**Table 3:** Accuracy of claims data to identify the Cesarean section and the difference between the date of claim data and the end date of pregnancy in EMRs.

						Relative Difference (The Date of Claim Data Minus the End Date of Pregnancy in EMRs) (d)				
		N of Subjects				0 Days	Within 3 Days	Within 7 Days	Within 14 Days	Within 30 Days
Code	Code Name	Claims	TP (%)	FP (%)	Unevaluable (%)	n (%) ^a^				
O820	Delivery by elective cesarean section (the earliest date) ^b^	16	15 (93.8)	1 (6.3)	0 (0.0)	3 (20.0)	4 (26.7)	6 (40.0)	11 (73.3)	14 (93.3)
O820	Delivery by elective cesarean section (the latest date) ^b^	16	15 (93.8)	1 (6.3)	0 (0.0)	3 (20.0)	5 (33.3)	7 (46.7)	12 (80.0)	14 (93.3)
K89800	Cesarean section (complexity fee addition)	262	262 (100.0)	0 (0.0)	0 (0.0)	260 (99.2)	262 (100.0)	262 (100.0)	262 (100.0)	262 (100.0)
K89801	Cesarean section (emergency cesarean section)	166	166 (100.0)	0 (0.0)	0 (0.0)	164 (98.8)	166 (100.0)	166 (100.0)	166 (100.0)	166 (100.0)
K89802	Cesarean section (elective cesarean section)	175	175 (100.0)	0 (0.0)	0 (0.0)	175 (100.0)	175 (100.0)	175 (100.0)	175 (100.0)	175 (100.0)

Abbreviations: EMRs, electronic medical records; TP, true positive; FP, false positive. ^a^ Regarding the relative difference, cases that were both true positive and available for “the end date of pregnancy in EMRs” are shown. Therefore, some numbers could have discrepancies between the number of true-positive cases and the numbers in the rows of the relative difference if some cases missed the end date of pregnancy in EMRs, even though the EMR outcome itself was identified. ^b^ Existing multiple entries in the same claims code for the same women; both the earliest and the latest dates were analyzed.

**Table 4:** Positive predictive values and the date difference between claims data and EMRs of algorithms for live birth, fetal death, and cesarean section.

				Relative Difference (The Date of Claim Data Minus the End Date of Pregnancy in EMRs) (d)				
				0 Days	Within 3 Days	Within 7 Days	Within 14 Days	Within 30 Days
Algorithm Name	Claims (N)	TP (n)	PPV (95% CI) (%)	n (%) ^a^				
**Live birth**								
Selected algorithm 1 (the earliest date) ^b,c^	786	771	98.1 (96.9–98.9)	632 (82.0)	730 (94.6)	742 (96.1)	757 (98.2)	770 (99.9)
Selected algorithm 1 (the latest date) ^b,c^	786	771	98.1 (96.9–98.9)	596 (77.3)	758 (98.4)	763 (99.1)	768 (99.6)	770 (99.9)
Selected algorithm 2 (the earliest date) ^b,d^	786	771	98.1 (96.9–98.9)	665 (86.3)	762 (98.8)	767 (99.5)	770 (99.9)	771 (100.0)
Selected algorithm 2 (the latest date) ^b,d^	786	771	98.1 (96.9–98.9)	597 (77.4)	759 (98.4)	764 (99.1)	768 (99.6)	770 (99.9)
**Fetal death**								
Selected algorithm 1 (the earliest date) ^b,e^	97	96	99.0 (94.4–100.0)	46 (48.9)	65 (69.1)	80 (85.1)	90 (95.7)	93 (98.9)
Selected algorithm 1 (the latest date) ^b,e^	97	96	99.0 (94.4–100.0)	78 (83.0)	84 (89.4)	90 (95.7)	91 (96.8)	93 (98.9)
Selected algorithm 2 (the earliest date) ^b,f^	93	92	98.9 (94.2–100.0)	64 (70.3)	77 (84.6)	85 (93.4)	89 (97.8)	90 (98.9)
Selected algorithm 2 (the latest date) ^b,f^	93	92	98.9 (94.2–100.0)	78 (85.7)	84 (92.3)	90 (98.9)	90 (98.9)	90 (98.9)
**Cesarean section**								
Selected algorithm 1 (the earliest date) ^b,g^	342	341	99.7 (98.4–100.0)	327 (95.9)	330 (96.8)	332 (97.4)	337 (98.8)	340 (99.7)
Selected algorithm 1 (the latest date) ^b,g^	342	341	99.7 (98.4–100.0)	339 (99.4)	341 (100.0)	341 (100.0)	341 (100.0)	341 (100.0)
Selected algorithm 2 ^h^	341	341	100.0 (98.9–100.0)	339 (99.4)	341 (100.0)	341 (100.0)	341 (100.0)	341 (100.0)

Abbreviations: CI, confidence interval; EMRs, electronic medical records; PPV, positive predictive value; TP, true positive. ^a^ Regarding the relative difference, cases that were both true positive and available for “the end date of pregnancy in EMRs” are shown. Therefore, some numbers could have discrepancies between the number of true-positive cases and the numbers in the rows of the relative difference if some cases missed the end date of pregnancy in EMRs, even though the EMR outcome itself was identified. ^b^ Existing multiple entries of claims codes in the algorithm for the same women; both the earliest and the latest dates were analyzed. ^c^ Codes in selected algorithm 1 for live birth were as follows: O601, O624, O654, O655, O669, O680, O683, O690, O700, O717, O721, O757, O800, O820, K89300, K89601, K89603, K89800, K89801, K89802, K90400, oxytocin injection, and dinoprost injection. ^d^ Codes in selected algorithm 2 for live birth were as follows: O601, O624, O654, O655, O669, O683, O690, O700, O717, O721, O800, K89300, K89601, K89603, K89800, K89801, K89802, K90400, oxytocin injection, and dinoprost injection. ^e^ Codes in selected algorithm 1 for fetal death were as follows: O008, O009, O010, O021, O034, O039, O049, O080, O081, O364, P95, K90901, K90902, K90920, and K91100. ^f^ Codes in selected algorithm 2 for fetal death were as follows: O008, O010, O034, O039, O049, O080, O364, P95, K90901, K90902, K90920, and K91100. ^g^ Included codes in selected algorithm 1 for cesarean section were as follows: O820, K89800, K89801, and K89802. ^h^ Included codes in selected algorithm 2 for cesarean section were as follows: K89800, K89801, and K89802.

## Data Availability

The data cannot be shared for privacy and ethical reasons.

## Supplementary Materials

The following supporting information can be downloaded at: https://www.mdpi.com/article/10.3390/ijerph19084864/s1, Table S1: Planned claims codes to identify birth-related outcomes; Table S2: Accuracy of claims data to identify miscarriage and the difference between the date of claim data and the end date of pregnancy in EMRs; Table S3: Accuracy of claims data to identify induced abortion and the difference between the date of claim data and the end date of pregnancy in EMRs; Table S4: Positive predictive values and the date difference between claims data and EMRs of algorithms for miscarriage and induced abortion.
