# Supplementary material for: Validity of Administrative Data for Identifying Birth-Related Outcomes with the End Date of Pregnancy in a Japanese University Hospital
Source: Int J Environ Res Public Health. 2022 Apr 16;19(8):4864. doi: 10.3390/ijerph19084864 (PMC9025717; doi:10.3390/ijerph19084864)
Supplement: Supplementary file 1 [file ijerph-19-04864-s001.zip › ijerph-1652183-supplementary.pdf]

Table S1. Planned[a] claims codes to identify birth-related outcomes.

Outcome		Code Type	Code
Live birth		ICD-10	O60-O75 (Complications of labour and delivery)[b], O80-O84 (Delivery), Z370 (Single live birth), Z372 (Twins, both liveborn), Z375 (Other multiple births, all liveborn)
		Medical procedures	K891 (Hysterotrachelectomy during delivery), K892 (Breech extraction), K893 (Vacuum extractions), K894 (Forceps delivery), K895 (Episiotomy and suture during delivery), K896 (Suture of perineal laceration during delivery), K897 (Suture of cervical laceration during delivery), K898 (Cesarean section), K904 (Obstetric hysterectomy-Porro's operation), J80 (Uterine dilation and labor induction)
		Medication	Oxytocin injection, Methylergometrine maleate injection, Dinoprost injection, Gemeprost suppository, Methylergometrine maleate tablet
Fetal death	Miscarriage/Stillbirth	ICD-10	O00 (Ectopic pregnancy), O01 (Hydatidiform mole), O02 (Other abnormal products of conception), O03 (Spontaneous abortion), O05 (Other abortion), O06 (Unspecified abortion), O08 (Complications following abortion and ectopic and molar pregnancy), O95 (Obstetric death of unspecified cause), O364 (Maternal care for intrauterine death), P95 (Fetal death of unspecified cause), Z371 (Single stillbirth), Z374 (Twins, both stillborn), Z377 (Other multiple births, all stillborn)
		Medical procedures	K899 (Destructive operations on the fetus), K909 (Surgical management of miscarriage), K90920 (Dilatation and curettage-missed abortion), K911 (Removal of hydatidiform mole), K912 (Ectopic pregnancy surgery)
	Induced abortion	ICD-10	O04 (Medical abortion), O07 (Failed attempted abortion)
		Medical procedures	K909 (Surgical management of miscarriage), K90920 (Dilatation and curettage-missed abortion)
Caesarean section		ICD-10	O820 (Delivery by elective cesarean section)
		Medical procedures	K898 (Cesarean section)

Abbreviations: ICD-10, International Classification of Diseases 10th revision

[a] Some claims codes could not be observed in this study because of the data availability during our study period, although we had initially planned to evaluate all the claims codes in this table.

[b] O600 (Preterm labour without delivery) was not included in the planned code for live birth.

Table S2. Accuracy of claims data to identify miscarriage and the difference between the date of claim data and the end date of pregnancy in EMRs

						Relative Difference (the date of claim data minus the end date of pregnancy in EMRs) (d)				
		N of subjects				0 days	Within 3 days	Within 7 days	Within 14 days	Within 30 days
**Code**	**Algorithm Name**	**Claims**	**TP (%)**	**FP (%)**	**Unevaluable (%)**			n (%)[a]		
O008	Other ectopic pregnancy	1	1 (100.0)	0 (0.0)	0 (0.0)	0 (0.0)	0 (0.0)	1 (100.0)	1 (100.0)	1 (100.0)
O009	Ectopic pregnancy, unspecified	2	0 (0.0)	1 (50.0)	1[b] (50.0)	0 (0.0)	0 (0.0)	0 (0.0)	0 (0.0)	0 (0.0)
O010	Classical hydatidiform mole (the earliest date)[c]	4	4 (100.0)	0 (0.0)	0 (0.0)	1 (25.0)	4 (100.0)	4 (100.0)	4 (100.0)	4 (100.0)
O010	Classical hydatidiform mole (the latest date)[c]	4	4 (100.0)	0 (0.0)	0 (0.0)	1 (25.0)	3 (75.0)	4 (100.0)	4 (100.0)	4 (100.0)
O011	Incomplete and partial hydatidiform mole	1	0 (0.0)	1 (100.0)	0 (0.0)	0 (0.0)	0 (0.0)	0 (0.0)	0 (0.0)	0 (0.0)
O021	Missed abortion	29	29 (100.0)	0 (0.0)	0 (0.0)	10 (35.7)	16 (57.1)	22 (78.6)	28 (100.0)	28 (100.0)
O028	Other specified abnormal products of conception	1	0 (0.0)	1 (100.0)	0 (0.0)	0 (0.0)	0 (0.0)	0 (0.0)	0 (0.0)	0 (0.0)
O034	Incomplete spontaneous abortion without complication	2	2 (100.0)	0 (0.0)	0 (0.0)	2 (100.0)	2 (100.0)	2 (100.0)	2 (100.0)	2 (100.0)
O039	Complete or unspecified spontaneous abortion without complication	5	5 (100.0)	0 (0.0)	0 (0.0)	3 (75.0)	4 (100.0)	4 (100.0)	4 (100.0)	4 (100.0)
O080	Genital tract and pelvic infection following abortion and ectopic and molar pregnancy	1	1 (100.0)	0 (0.0)	0 (0.0)	1 (100.0)	1 (100.0)	1 (100.0)	1 (100.0)	1 (100.0)
O081	Delayed or excessive haemorrhage following abortion and ectopic and molar pregnancy	1	1 (100.0)	0 (0.0)	0 (0.0)	0 (0.0)	0 (0.0)	0 (0.0)	0 (0.0)	1 (100.0)
O364	Maternal care for intrauterine death (the earliest date)[c]	8	6 (75.0)	2[d] (25.0)	0 (0.0)	0 (0.0)	1 (16.7)	5 (83.3)	6 (100.0)	6 (100.0)
O364	Maternal care for intrauterine death	8	6 (75.0)	2[d] (25.0)	0 (0.0)	1 (16.7)	2 (33.3)	6 (100.0)	6 (100.0)	6 (100.0)

	(the latest date)[c]									
P95	Fetal death of unspecified cause	1	1 (100.0)	0 (0.0)	0 (0.0)	1 (100.0)	1 (100.0)	1 (100.0)	1 (100.0)	1 (100.0)
K90901	Surgical management of miscarriage (until 11 weeks)	35	35 (100.0)	0 (0.0)	0 (0.0)	35 (100.0)	35 (100.0)	35 (100.0)	35 (100.0)	35 (100.0)
K90902	Surgical management of miscarriage (from 12 weeks to 21 weeks)	1	1 (100.0)	0 (0.0)	0 (0.0)	1 (100.0)	1 (100.0)	1 (100.0)	1 (100.0)	1 (100.0)
K90920	Dilatation and curettage (missed abortion)	27	3 (11.1)	24 (88.9)	0 (0.0)	2 (66.7)	2 (66.7)	2 (66.7)	2 (66.7)	2 (66.7)
K91100	Removal of hydatidiform mole	6	6 (100.0)	0 (0.0)	0 (0.0)	6 (100.0)	6 (100.0)	6 (100.0)	6 (100.0)	6 (100.0)

Abbreviations: EMRs, electronic medical records; TP, true positive; FP, false positive

[a] Regarding the relative difference, cases that were both true positive and available for "the end date of pregnancy in EMRs" are shown. Therefore, some numbers could have discrepancies between the number of true positive cases and the numbers in the rows of the relative difference, if some cases missed the end date of pregnancy in EMRs, even though the EMR outcome itself was identified.

[b] One case in O009 was undetermined as whether miscarriage or stillbirth.

[c] Existing multiple entries in the same claims code for the same women; both the earliest and the latest dates were analyzed.

[d] Two cases in O364 were identified as stillbirth instead of miscarriage.

Table S3. Accuracy of claims data to identify induced abortion and the difference between the date of claim data and the end date of pregnancy in EMRs

						Relative Difference (the date of claim data minus the end date of pregnancy in EMRs) (d)				
		N of subjects				0 days	Within 3 days	Within 7 days	Within 14 days	Within 30 days
**Code**	**Code Name**	**Claims**	**TP (%)**	**FP (%)**	**Unevaluable (%)**			**n (%)[a]**		
O049	Medical abortion, complete or unspecified, without complication (the earliest date)[b]	22	21 (95.5)	0 (0.0)	1 (4.5)	7 (33.3)	15 (71.4)	17 (81.0)	20 (95.2)	21 (100.0)
O049	Medical abortion, complete or unspecified, without complication (the latest date)[b]	22	21 (95.5)	0 (0.0)	1 (4.5)	8 (38.1)	17 (81.0)	19 (90.5)	21 (100.0)	21 (100.0)
K90901	Surgical management of miscarriage (until 11 weeks)	35	0 (0.0)	35 (100.0)	0 (0.0)	0 (0.0)	0 (0.0)	0 (0.0)	0 (0.0)	0 (0.0)
K90902	Surgical management of miscarriage (from 12 weeks to 21 weeks)	1	0 (0.0)	1 (100.0)	0 (0.0)	0 (0.0)	0 (0.0)	0 (0.0)	0 (0.0)	0 (0.0)
K90920	Dilatation and curettage (missed abortion)	27	24 (88.9)	3 (11.1)	0 (0.0)	24 (100.0)	24 (100.0)	24 (100.0)	24 (100.0)	24 (100.0)

Abbreviations: EMRs, electronic medical records; TP, true positive; FP, false positive

[a] Regarding the relative difference, cases that were both true positive and available for "the end date of pregnancy in EMRs" are shown. Therefore, some numbers could have discrepancies between the number of true positive cases and the numbers in the rows of the relative difference, if some cases missed the end date of pregnancy in EMRs, even though the EMR outcome itself was identified.

[b] Existing multiple entries in the same claims code for the same women; both the earliest and the latest dates were analyzed.

Table S4. Positive predictive values and the date difference between claims data and EMRs of algorithms for miscarriage and induced abortion

				Relative Difference (the date of claim data minus the end date of pregnancy in EMRs) (d)				
				0 days	Within 3 days	Within 7 days	Within 14 days	Within 30 days
**Algorithm Name**	**Claims (N)**	**TP (n)**	**PPV (95% CI) (%)**			**n (%)[a]**		
**Miscarriage**								
Selected algorithm 1 (the earliest date)[b,c]	54	54	100.0 (93.4–100.0)	28 (53.8)	38 (73.1)	45 (86.5)	51 (98.1)	52 (100.0)
Selected algorithm 1 (the latest date)[b,c]	54	54	100.0 (93.4–100.0)	46 (88.5)	47 (90.4)	49 (94.2)	51 (98.1)	52 (100.0)
Selected algorithm 2 (the earliest date)[b,d]	50	50	100.0 (92.9–100.0)	44 (89.8)	48 (98.0)	49 (100.0)	49 (100.0)	49 (100.0)
Selected algorithm 2 (the latest date)[b,d]	50	50	100.0 (92.9–100.0)	46 (93.9)	47 (95.9)	49 (100.0)	49 (100.0)	49 (100.0)
**Induced abortion**								
Selected algorithm 1 (the earliest date)[b,e]	37	33	89.2 (74.6–97.0)	19 (57.6)	27 (81.8)	29 (87.9)	32 (97.0)	33 (100.0)
Selected algorithm 1 (the latest date)[b,e]	37	33	89.2 (74.6–97.0)	28 (84.8)	33 (100.0)	33 (100.0)	33 (100.0)	33 (100.0)
Selected algorithm 2 (the earliest date)[b,f]	37	33	89.2 (74.6–97.0)	19 (57.6)	27 (81.8)	29 (87.9)	32 (97.0)	33 (100.0)
Selected algorithm 2 (the latest date)[b,f]	37	33	89.2 (74.6–97.0)	28 (84.8)	33 (100.0)	33 (100.0)	33 (100.0)	33 (100.0)

Abbreviations: CI, confidence interval; EMRs, electronic medical records; PPV, positive predictive value; TP, true positive

[a] Regarding the relative difference, cases that were both true positive and available for "the end date of pregnancy in EMRs" are shown. Therefore, some numbers could have discrepancies between the number of true positive cases and the numbers in the rows of the relative difference, if some cases missed the end date of pregnancy in EMRs, even though the EMRs outcome itself was identified.

[b] Existing multiple entries of claims codes in the algorithm for the same women; both the earliest and the latest dates were analyzed.

[c] Codes in selected algorithm 1 for miscarriage were as follows: O008, O010, O021, O034, O039, O080, O081, P95, K90901, K90902, and K91100.

[d] Codes in selected algorithm 2 for miscarriage were as follows: O008, O010, O034, O039, O080, P95, K90901, K90902, and K91100.

[e] Codes in selected algorithm 1 for induced abortion were as follows: O049 and K90920.

[f] Codes in selected algorithm 2 for induced abortion were the same as selected algorithm 1.
